# Oocyte–Targeted Deletion Reveals That Hsp90b1 Is Needed for the Completion of First Mitosis in Mouse Zygotes

**DOI:** 10.1371/journal.pone.0017109

**Published:** 2011-02-15

**Authors:** Christophe Audouard, Florent Le Masson, Colette Charry, Zihai Li, Elisabeth S. Christians

**Affiliations:** 1 Centre de Biologie du Développement, Université Toulouse 3 - Paul Sabatier, UPS, UMR 5547, Toulouse, France; 2 Division of Basic Sciences, Hollings Cancer Center, Department of Microbiology and Immunology, Medical University of South Carolina, Charleston, South Carolina, United States of America; McGill University, Canada

## Abstract

**Background:**

Hsp90b1 is an endoplasmic reticulum (ER) chaperone (also named Grp94, ERp99, gp96,Targ2, Tra-1, Tra1, Hspc4) (MGI:98817) contributing with Hspa5 (also named Grp78, BIP) (MGI:95835) to protein folding in ER compartment. Besides its high protein expression in mouse oocytes, little is known about Hsp90b1 during the transition from oocyte-to-embryo. Because the constitutive knockout of Hsp90b1 is responsible for peri-implantation embryonic lethality, it was not yet known whether Hsp90b1 is a functionally important maternal factor.

**Methodology/Findings:**

To circumvent embryonic lethality, we established an oocyte-specific conditional knockout line taking advantage of the more recently created floxed Hsp90b1 line (*Hsp90b1^flox^*, MGI:3700023) in combination with the transgenic mouse line expressing the cre recombinase under the control of zona pellucida 3 (ZP3) promoter (Zp3-cre, MGI:2176187). Altered expression of Hsp90b1 in growing oocytes provoked a limited, albeit significant reduction of the zona pellucida thickness but no obvious anomalies in follicular growth, meiotic maturation or fertilization. Interestingly, mutant zygotes obtained from oocytes lacking Hsp90b1 were unable to reach the 2-cell stage. They exhibited either a G2/M block or, more frequently an abnormal mitotic spindle leading to developmental arrest. Despite the fact that Hspa5 displayed a similar profile of expression as Hsp90b1, we found that HSPA5 and HSP90B1 did not fully colocalize in zygotes suggesting distinct function for the two chaperones. Consequently, even if HSPA5 was overexpressed in *Hsp90b1* mutant embryos, it did not compensate for HSP90B1 deficiency. Finally, further characterization of ER compartment and cytoskeleton revealed a defective organization of the cytoplasmic region surrounding the mutant zygotic spindle.

**Conclusions:**

Our findings demonstrate that the maternal contribution of Hsp90b1 is critical for the development of murine zygotes. All together our data indicate that Hsp90b1 is involved in unique and specific aspects of the first mitosis, which brings together the maternal and paternal genomes on a single spindle.

## Introduction

Analysis of the mouse oocyte proteome identified several chaperones among the proteins particularly enriched in the female gametes [1], [2]. Chaperones are proteins, which transiently interact with other polypeptides to contribute to their appropriate folding or to their degradation if they are abnormal. This important group of proteins, which prevent severe dysfunction in most cellular processes includes the heat shock proteins (HSPs). HSPs are encoded by genes characterized by their inducible expression triggered by various stress including proteotoxic stress such as heat shock. Two members of the Hsp90 family, the cytoplasmic Hsp90aa1 and Hsp90b1, the reticulum endoplasmic paralog of Hsp90aa1, were reported to be the 4^th^ and 7^th^ most abundant proteins in the oocytes, respectively [2]. In our previous work, we showed that the activity of HSP90AA1 (also named Hsp90alpha, Hsp86, Hsp4, Hspca, Hspc1) was important to prevent meiotic defects (G2/M delay, abnormal meiotic cytokinesis) [3].

Here we focused our attention on Hsp90b1 (also named Grp94, Gp96) as a potential gene with maternal effect due to its high expression in oocytes. HSP90B1 is located in the endoplasmic reticulum (ER) where numerous proteins translocate and require adequate folding. If misfolded proteins accumulate in ER, this induces a so-called ER stress and triggers the unfolded protein response (UPR) which induces the expression of several genes including ER chaperones such Hsp90b1 or Hspa5 [4]. Very little is know about this defense mechanism and the role of ER chaperones in oocytes and early embryos. Hsp90b1 knockout is lethal by day 7 post-fertilization due to failure to establish mesoderm germ cell layer [5], [6]. Using embryonic stem cells derived from the mutant embryos before they died, Wanderling and collaborators showed that HSP90B1 was required for muscle differentiation in relation with its capacity to chaperone neoynthesized IGF2 and subsequently allow IGF2 secretion and paracrine action [6]. Hence, because the constitutive knockout was embryonic lethal and the cellular model was inappropriate to study the role of Hsp90b1 in female gametes and early preimplantation embryos, it was necessary to use a conditional gene targeting approach.

We took advantage of the line generated by Yang et al. (2007) [7] to create an oocyte specific deletion of Hsp90b1 using the Zp3-cre transgenic line [8], [9]. Our work revealed that oocyte specific loss-of-function of Hsp90b1 was not essential for meiosis or fertilization but impaired the progression of mutant zygotes during their first cell cycle. At the molecular level, we found indications that Hsp90b1 and Hspa5 were coregulated during the transition from egg to embryo but Hspa5 could not compensate for the lack of Hsp90b1. At the cellular level, absence of HSP90B1 induced ER and cytoskeleton disorganization during the first embryonic M phase. All together these data highlight the importance of ER chaperones during the very first steps of development.

## Results

### ER chaperones Hsp90b1 and Hspa5 are coregulated during the oocyte-to-embryo transition

**Figure 1:**
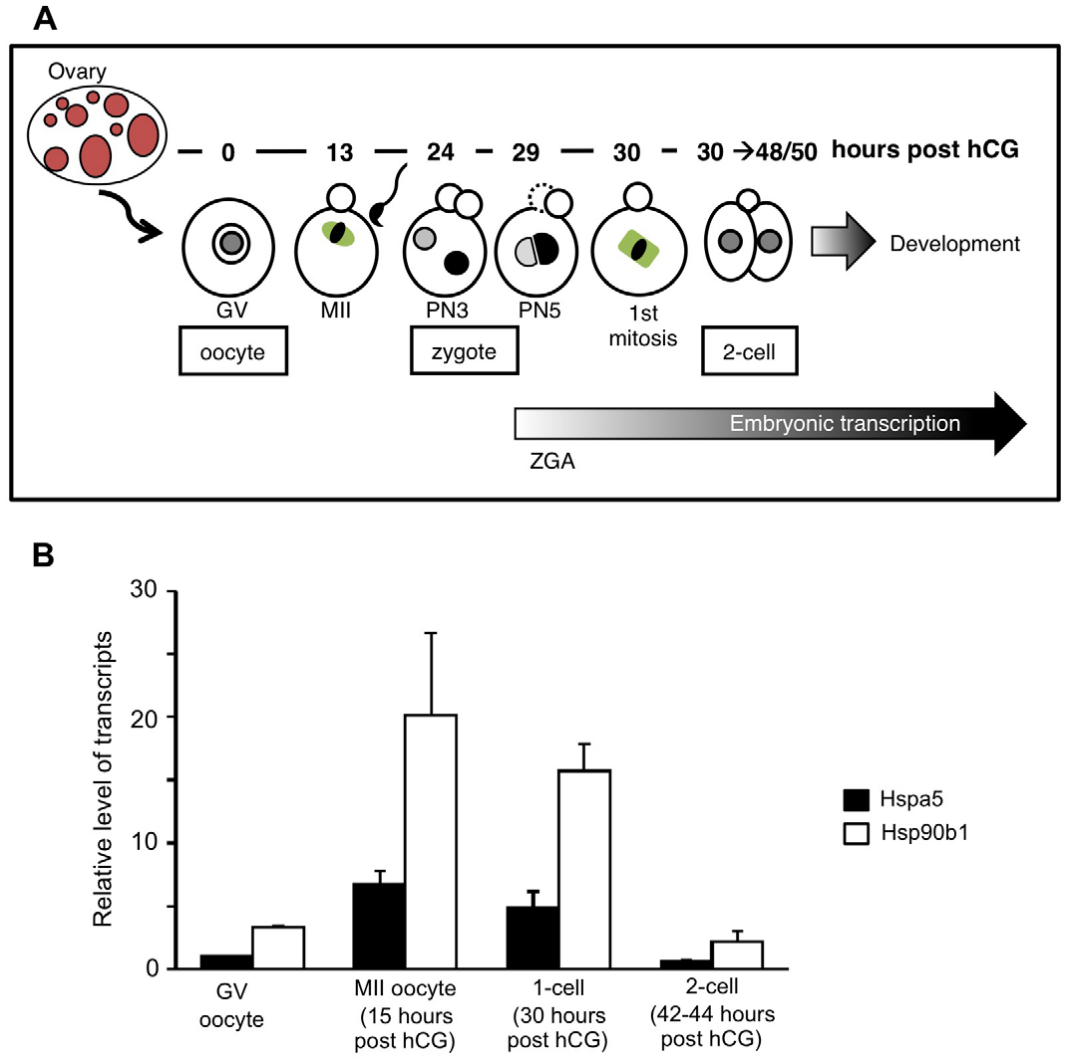
Transition from oocyte-to-embryo and expression of ER chaperones. (**A**) Adult ovary contains oocytes included in follicles which grow from primordial to primary, secondary and antral stages. Puncture of antral follicles releases fully grown oocytes at the germinal vesicle stage (GV oocytes). In culture, or in vivo following hormonal stimulation, GV oocytes resume meiosis to progress to the metaphase II stage (MII). MII oocytes can be fertilized and within few hours, the newly formed zygotes contain two parental pronuclei, which develop and migrate towards each other. During the G2 phase of the first cell cycle, the zygotic genome activation (ZGA) occurs producing the first embryonic transcripts. Then the zygotes enter the first mitosis and cleave to form 2-cell stage embryos. When females are superovulated, ovulation occurs at about 13 hours post hCG and the timing of early embryonic development can be subsequently described following the number of hours after injection of human chorionic gonadotrophin (hours post hCG). (**B**) RTqPCR was used to determine the level of Hspa5 and Hsp90b1 transcripts during the transition from oocyt-to-embryo in WT samples. Relative quantities of mRNA were normalized against the quantity of ribosomal S16 transcripts and Hspa5 transcript level in WT oocytes (GV) was arbitrarily given the value 1.

HSP90b1 was reported to be one of the most abundant protein in oocytes [2]. However very few is known about the expression of this chaperone during the oocyte-to-embryo transition whose developmental steps are presented in Figure 1A.

We used RTqPCR to analyze the level of Hsp90b1 and Hspa5 transcripts because it was previously described in somatic or cancer cells that Hspa5, a related major ER chaperone is coordinately regulated with Hsp90b1 [5], [10]. Figure 1B shows that both chaperones exhibit a paralleled profile with a similar increased amount of transcripts from GV to MII oocytes and then a decrease up to the 2-cell stage. We also observed a higher level of Hsp90b1 than Hspa5 transcripts as it could be expected from proteomic data [2]. These data reinforced the importance of the maternal contribution made by these two ER chaperones.

### Altered expression of Hsp90b1 is compatible with oogenesis and meiotic maturation

From the high level of Hsp90b1 expression it could be hypothesized that HSP90b1 is needed during oogenesis and/or early embryonic development. To test this hypothesis, we generated an oocyte-specific deletion of Hsp90b1 using the transgenic mouse line expressing the cre recombinase under Zp3 promoter (Zp3-cre) and the floxed Hsp90b1 line (*Hsp90b1^flox^*) [7], [8].

**Figure 2:**
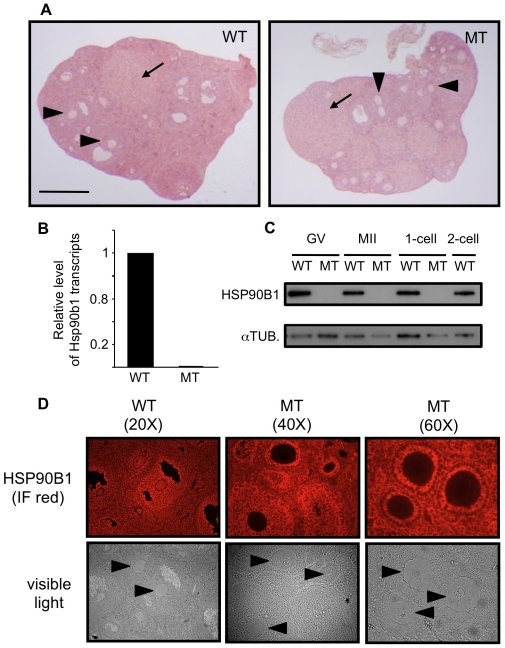
Ovary histology and Hsp90b1 expression. (**A**) Histological sections of wild-type (WT) and Zp3-cre*; Hsp90b1^flox/flox^* (MT) ovary were HE stained WT and MT sections contain follicles at different stages including visible oocytes (arrow head) and corpus luteus (arrow). Scale bar: 0.5 mm. (**B**) RTqPCR was used to determine the level of Hsp90b1 transcripts in WT and MT fully grown oocytes (GV stage). Relative quantities of mRNA were normalized against the quantity of ribosomal S16 transcripts and Hsp90b1 transcript level in WT oocytes was arbitrarily given the value 1. (**C**) Western blot analysis of HSP90B1 protein content in fully grown (GV), metaphase II (MII) oocytes and in 1-cell, 2-cell embryos. Samples collected from WT and MT females are shown as indicated. Membranes were reprobed with anti alpha-tubulin (as aTUB.) as loading control. (**D**) HSP90B1 immunodetection was performed on ovary histological sections. Left panels (WT) show HSP90B1 expression in both somatic cells and oocytes at various follicular stages. Middle panels (MT) illustrate the oocyte specific loss of HSP90B1 in MT sample. Right panels (MT) show 3 primary follicles containing an oocyte section. HSP90B1 is strongly immunodetected in granulosa cells while it is barely detectable in oocyte cytoplasm. Arrowheads indicate GV oocytes included in the sections.

To determine whether this strategy was successful and whether Hsp90b1 deletion had consequences during oogenesis, we first performed histological analysis and HSP90B1 immunodetection on ovary sections in wild-type (WT) and mutant females (Zp3-cre; *Hsp90b1^flox/flox^*, indicated thereafter as MT). Ovary histomorphology appeared grossly normal in MT females with the presence of all the follicular stages and corpus luteum as in WT females (Figure 2A).

Second, we compared the level of Hsp90b1 expression in WT and MT samples. RTqPCR was performed with fully-grown (GV) oocytes (Figure 2B). The level of transcripts in MT oocytes was below detection but due to the slow turn-over of HSP90B1 protein [7], absence of transcripts could not be merely correlated with the loss of HSP90B1 in those oocytes. Western blot experiments were then carried out with several samples encompassing the oocyte-to-embryo transition (Figure 2C). There was no HSP90B1 signal visible in MT extracts while alpha-tubulin was similarly detected in MT as in WT samples.

Third we used fluorescent immunodetection (and immunohistochemistry, data not shown) to determine HSP90B1 presence and localization in WT and MT ovary sections. Immunostaining revealed that this chaperone was normally expressed in oocytes as well as in granulosa cells (Figure 2D, left panels). The strong signal exhibited by HSP90B1 was lost in oocytes where Zp3-cre had induced a complete and specific recombination of Hsp90b1 starting at the primary follicle stage as expected from the regulation exerted by the Zp3 promoter (Figure 2D, middle and right panels) [8], [9].

**Figure 3:**
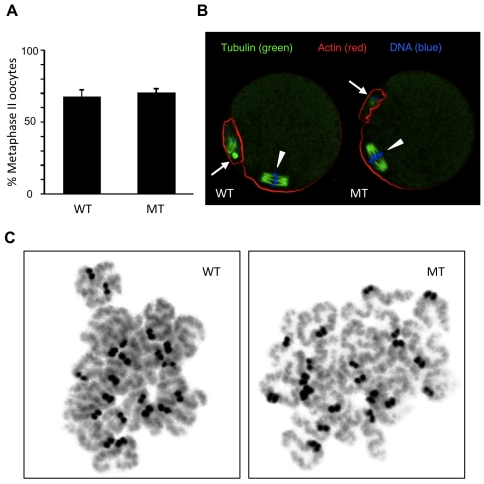
HSP90B1 is not essential for meiotic maturation. (**A**) Mean percentage of GV (WT and MT) oocytes which reached MII stage after in vitro culture. (**B**) MII oocytes were processed for microtubule, actin and DNA staining. Arrowheads indicate the MII chromosomes and arrows show the first polar body resulting from the first meiotic division (see Figure 1A). (**C**) Chromosome spreads were prepared using MII (WT and MT) oocytes. They show chromosomes with 2 chromatids as expected before the 2^nd^ meiotic division.

Consistent with the overall normal ovarian histology, the same number of fully-grown oocytes (GV oocyte, Figure 1A) could be obtained by puncture of MT ovaries (data not shown). In vitro meiotic maturation was carried out by culturing the GV oocytes as previously described [3]. Figure 3A shows that the percentage of WT and MT oocytes, which extruded the first polar body and reached MII was not significantly different. WT and MT mature oocytes were processed for immunofluorescence in order to examine the MII spindle, which appeared to be morphologically normal (Figure 3B). MII chromosome spreads were analyzed and no visible anomaly could be found (Figure 3C).

In conclusion from those data, oocyte growth and meiotic maturation could be achieved while HSP90B1 expression had been severely altered from the beginning of growth phase.

### Loss of HSP90B1 during oogenesis perturbed zona pellucida formation

**Figure 4:**
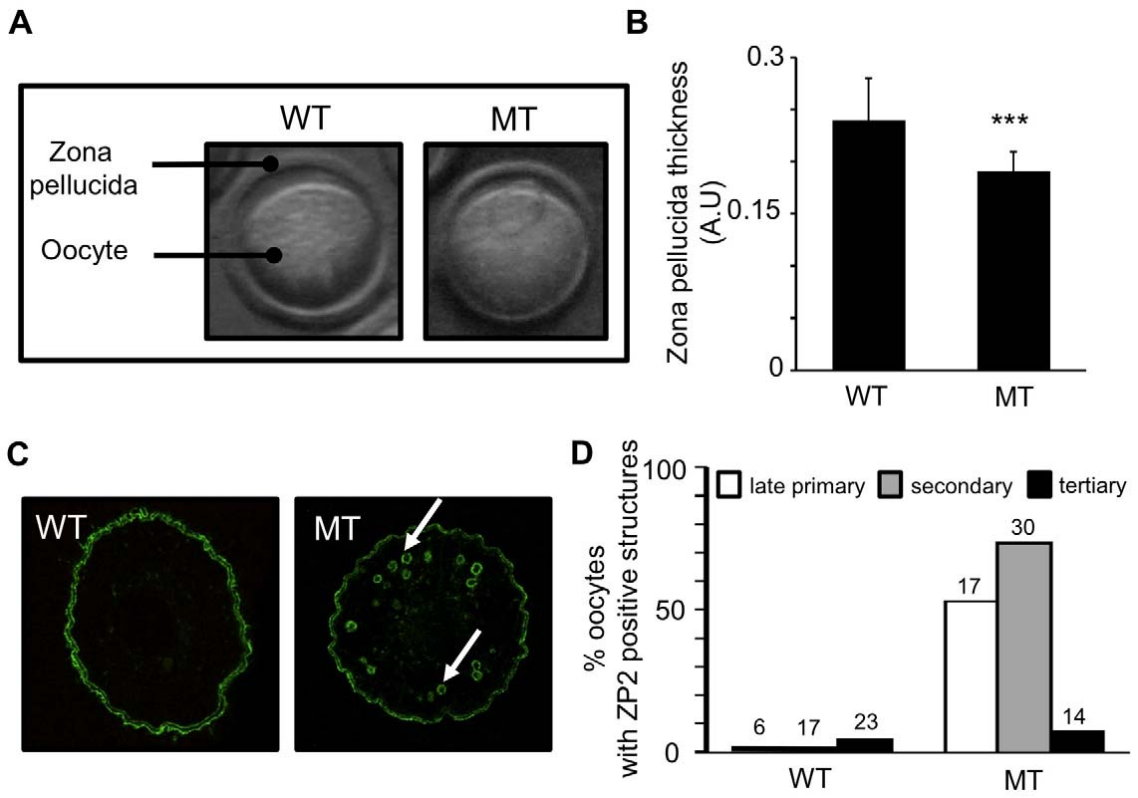
Thinner zona pellucida in HSP90B1 deficient oocytes. (**A**) Representative images of wild-type (WT) and mutant (MT, obtained from Zp3-cre*; Hsp90b1^flox/flox^* females) ovulated oocytes showing that zona pellucida is thinner in mutant oocytes. (**B**) Ratio between thickness of zona pellucida and oocyte diameter was calculated and indicated in arbitrary unit (A.U.). Mutant zona pellucida (n = 99) were significantly thinner than wild type ones (n = 36). p<0.001. (**C**) Histological sections of adult ovaries were prepared for immunofluorescence against ZP2 and representative examples of WT and MT oocytes from secondary follicles are shown. Arrows indicate the ZP2 positive structures in mutant oocytes.(**D**) Percentage of WT and MT oocytes containing ZP2 positive structures at late primary, secondary and tertiary follicular stages. Numbers indicated above the bar correspond to the number of follicles for each category.

When collecting immature and mature oocytes, we noticed that they were surrounded by a significantly thinner zona pellucida (Figure 4A, B). As the zona pellucida is synthesized during oocyte growth phase, we immunostained ovarian sections with antibodies against the three ZP glycoproteins. Probably, for technical reasons, we could not obtain good staining with ZP1 and ZP3 antibodies in WT ovaries. Nevertheless we had better results with anti-ZP2 labeling, which revealed the presence of round and strongly stained structures in MT sections. Representative images of WT and MT secondary follicles are displayed in Figure 4C. We further quantified the number of oocytes exhibiting those structures from late primary to tertiary follicular stages. The graph presented in Figure 4D shows that those structures were rarely observed in WT oocytes (0/17 oocytes at the secondary follicular stage) but were frequently observed in MT oocytes (22/30 oocytes at the secondary follicular stage). Such structures could suggest that, in absence of HSP90B1, the trafficking of ZP glycoproteins is altered but not abolished, providing an explanation for the reduced thickness of the zona pellucida.

### Hsp90b1, a gene with maternal effect

**Figure 5:**
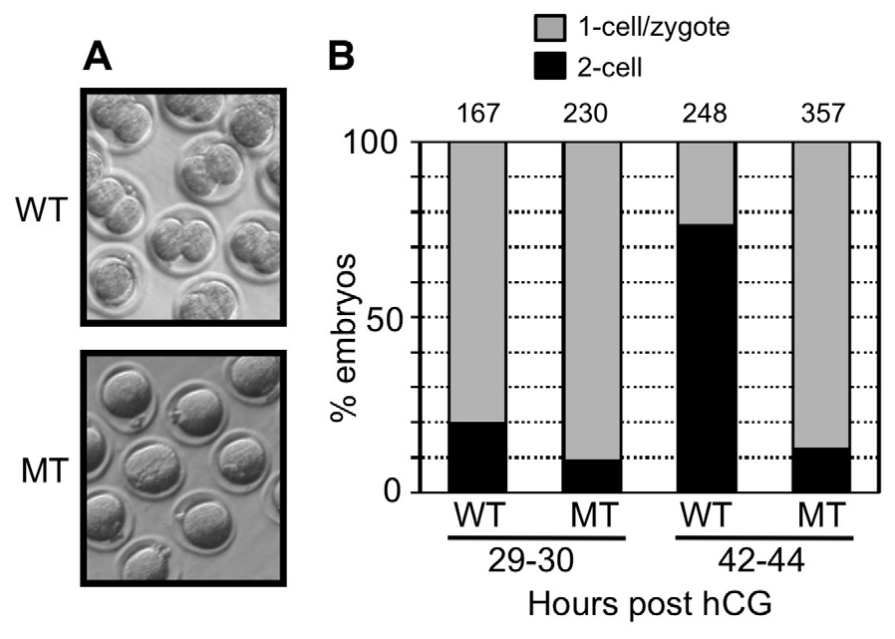
Maternal Hsp90B1 is needed to obtain 2-cell embryos. (**A**) Representative picture of WT and MT embryos at 42–44 hours post hCG. As expected, WT embryos were at the 2-cell stage while most MT embryos remained blocked at the one-cell stage. (**B**) Respective percentage of 1-cell and 2-cell embryos was determined at 29–30 (Chi square, p<0.01) and 42–44 (Chi square, p<0.0001) hours post hCG. As shown in Figure 1A, the first cleavage starts about 29–30 hours post hCG and should be completed by 42–44 hours post hCG. The number of analyzed embryos are indicated above the bars.

After mating with WT males, litters of MT females were significantly smaller than those obtained from WT females (3.3±0.45 pups instead of 7.3±0.7 pups, respectively) (Table 1). The pups born from MT females mated with WT males were genotyped and they were *Hsp90b1^del/+^* as expected. Thus the WT allele received from the WT father was not able to restore a normal reproductive phenotype as shown by the size of the litters produced by MT females. This led us to conclude that the MT phenotype was dependent on the genotype of the mother and that Hsp90b1 was a gene with maternal effect [11]. To determine the origin of the smaller litters produced by MT females, we focused our attention on the early steps of embryonic development that are known to be under maternal control [11]. Analysis performed on in vivo development showed a reduced competence of MT embryos to develop up to the blastocyst stage (Table 1). After superovulation and in vivo fertilization we obtained the same number of fertilized MT and WT zygotes exhibiting two visible pronuclei (26.7±1.9 versus 27.1±2 zygotes, p = 0.89). However after they were cultured in vitro overnight (42–44 hours post hCG), most of the MT zygotes (82%) remained blocked at the one-cell stage instead of being cleaved to the 2-cell stage as WT embryos (Figure 5, Table 1) indicating that Hsp90b1 was needed to successfully accomplish the first cleavage of embryonic development.

**Table 1:** Development of embryos obtained by crossing *Zp3-cre; Hsp90b1^flox/flox^* (MT) or control females with wild-type (WT) males.

Experimental conditions for embryo production and development	Type of embryos according to mother genotype (N = females, n = embryos)	
	MT	Control
**natural ovulation & fertilization:**		
- in vivo development:		
nb pups per female	3.3±0.45	7.3±0.7
	(N = 21; n = 70)	(N = 8; n = 59)
- in vivo 1 st cleavage followed by in vitro culture:		
% Morula/Blastocyst	30±0.117	76.8±0.07
	(N = 5; n = 36)	(N = 8; n = 53)
- in vitro 1st cleavage:		
% 2-cell	8.7±0.05	100
	(N = 11; n = 106)	(N = 3; n = 23)
**superovulation & fertilization:**		
- in vitro 1st cleavage:		
% 2-cell	12.2±0.02	75±0.05
	(N = 17, n = 357)	(N = 13; n = 248)

### Progression through first mitosis is severely hampered in mutant embryos

**Figure 6:**
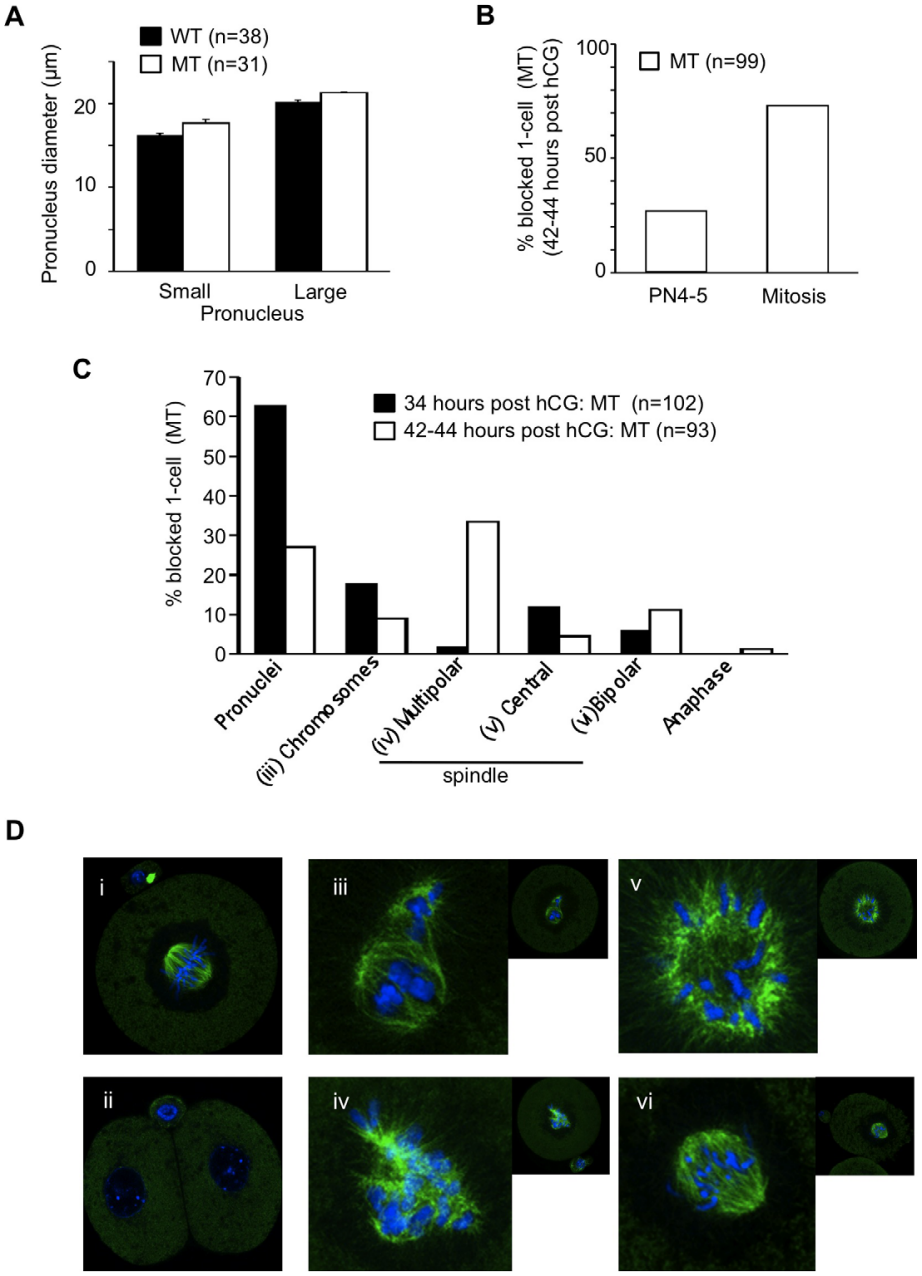
G2/M block and abnormal mitosis in mutant embryos. (**A**) The size of both maternal (expected to be smaller) and paternal (expected to be larger) pronuclei was measured in one–cell WT and MT embryos at 29–30 hours post hCG in order to determine their progression in the G2 phase of the first cell cycle. No significant difference was found between WT and MT pronuclei. (**B**) One-cell mutant embryos were analyzed at 42–44 hours post hCG to determine the percentage of embryos blocked at the G2/M transition or during mitosis. (**C**) One-cell mutant embryos were analyzed at 34 and 42–44 hours post-hCG and classified according to their nuclear or spindle morphology. NB: WT embryos had mostly reached the 2-cell stage at 34 hours posthCG (see Figure 5). (**D**) Tubulin (green) and DNA (blue) fluorescent staining of WT and MT embryos. Representative images of mitotic (i) and 2-cell (ii) WT embryos. Spindle morphologies of MT embryos are illustrated from iii to vi: (iii) two groups of chromosomes; (iv) multipolar spindle; (v) central organization of the spindle with peripherical chromosomes; (vi) a ‘normal’ bipolar spindle. Insets show the corresponding zygote.

To better identify at which developmental step MT embryos were arrested, we analyzed the progression of the WT and MT embryos during the first cell cycle. This can be determined by the size and position of both pronuclei corresponding to PN1 to PN5 stages (see Figure 1A) [12]. We measured the diameter of maternal (small) and paternal (large) pronuclei in WT and MT zygotes. We did not find any significant difference in pronucleus development between WT and MT zygotes (Figure 6A).

Then we collected MT embryos blocked at 1-cell stage at 42–44 hours post hCG and stained them to label DNA and tubulin in order to determine at which stage of the cell cycle they were arrested. Figure 6B shows that 25% of those MT embryos had remained at the pronuclear stage while the others were arrested during mitosis. Thus, based on these data, we could conclude that the progression of MT zygotes during the first cell cycle did not exhibit any visible anomaly until the first embryonic G2/M transition.

Figure 6C presents the percentage of the different morphologies exhibited by the 1-cell MT embryos observed at 34 or 42–44 hours post hCG and illustrated in Figure 6D. Figure 6D includes two panels (i-ii) to present the normal (WT) appearance of mitotic zygotes (34 h post hCG) and 2-cell embryos (42–44 h post hCG). MT embryos arrested during the first mitosis showed dispersed groups of chromosomes (Figure 6D, iii), multipolar spindle (Figure 6D, iv), centrally focused microtubules with peripherical attached chromosomes (Figure 6D, v) and bipolar spindle (Figure 6D, vi). Although multipolar spindle was the most striking and frequent observed anomaly (up to 33% at 42–44 hours post hCG) (Figure 6C, D, iv), it is worth noting that nearly normal-bipolar spindles were found in arrested mutant embryos suggesting that progression through mitosis was not simply altered due to spindle disorganization.

### Deletion of HSP90b1 modified HSPa5 expression but not localization

**Figure 7:**
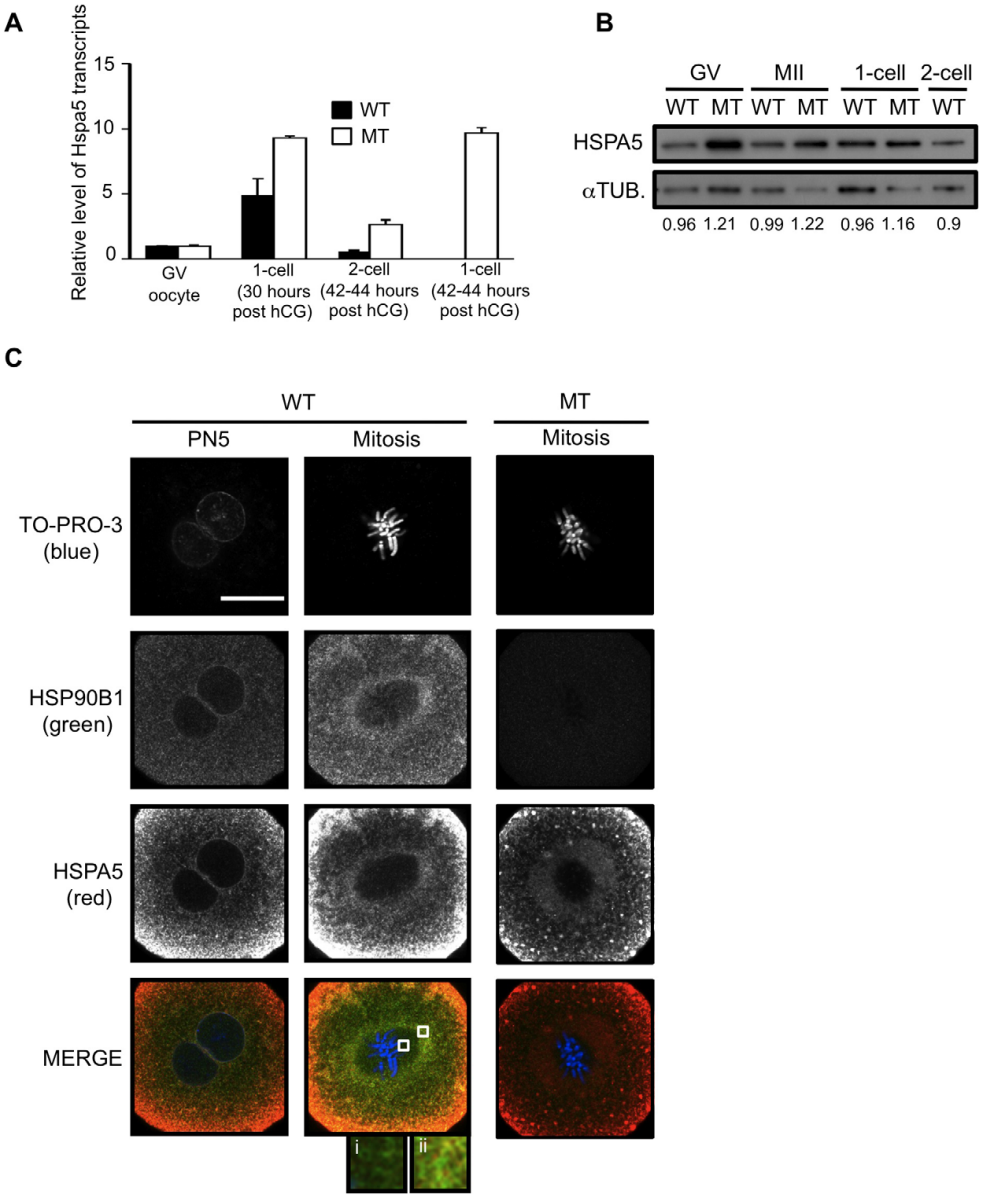
HSPA5 expression but not localization is modified in MT embryos. (**A**) RTqPCR was used to determine the level of Hspa5 transcripts in WT and MT samples: GV oocyte, ovulated MII oocytes, 1-cell or zygote (30 hours post hCG) and 2-cell (42–44 hours post hCG) embryos. Relative quantities of mRNA were normalized against the quantity of ribosomal S16 transcripts and Hspa5 transcript level in GV oocytes was arbitrarily given the value 1. (**B**) HSPA5 was immunodetected by Western blot using WT and MT samples as indicated. Experimental procedure was similar as in Figure 2C. The number written below each lane corresponds to the relative densitometric value (HSPA5/alphaTUB.). (**C**) WT embryos (pronuclear -PN5- and mitotic 1-cell stages) and MT mitotic zygotes were prepared for immunofluorescence as described in materials and methods. Representative images show that ER chaperones HSPA5 and HSP90B1 did not completely colocalize. HSP90B1 was found within the spindle structure but not HSPA5 as shown in inset (i). HSPA5 and HSP90B1 were both present in the granular region surrounding the spindle but higher magnification (inset ii) showed that even in this region where both ER chaperones were present, they were not fully colocalized. HSPA5 seemed to be more abundant in the cortex region of the zygote. HSPA5 staining was not visibly modified in MT embryos.

As described above, Hsp90b1 and Hspa5 are known to be often coregulated and we found that they exhibited the same profile of expression during the transition from egg-to-embryo (Figure 1B). Therefore the mutation of Hsp90b1 could modify the expression of Hspa5. Figure 7(A–B) presents the results obtained by RTqPCR and Western blot. Hspa5 transcripts accumulated significantly in MT embryos but not in fully grown oocytes (GV) (Figure 7A). There was no direct correlation with HSPA5 protein level which was increased to a similar extent in MT oocytes as in embryos (Figure 7B). All together our data (Figures 1B, 7A, 7B) indicated that ER chaperones were regulated at both transcriptional and translational levels in oocytes and embryos leading to some compensatory mechanism to sustain the level of ER chaperone proteins.

Having identified the developmental stage requiring HSP90B1, we were then interested in the localization of both ER chaperones to better understand ER function in zygotes. Figure 7C reveals that HSP90B1 and HSPA5 did not fully colocalize in WT zygotes. In mitotic zygotes, HSP90B1 could be detected within the spindle area but not HSPA5. In contrast both HSP90B1 and HSPA5 were found in the ER accumulation around the spindle. HSPA5 staining was also more pronounced in the cortical ER compartment. In MT mitotic embryos which did not contain HSP90B1 protein any more, HSPA5 remained similarly localized as in WT (Figure 7C, right panels). This suggested specific functions for both chaperones and could explain why overexpressed HSPA5 did not rescue MT embryos.

### ER compartment and cytoskeleton were disorganized around mutant spindles

**Figure 8:**
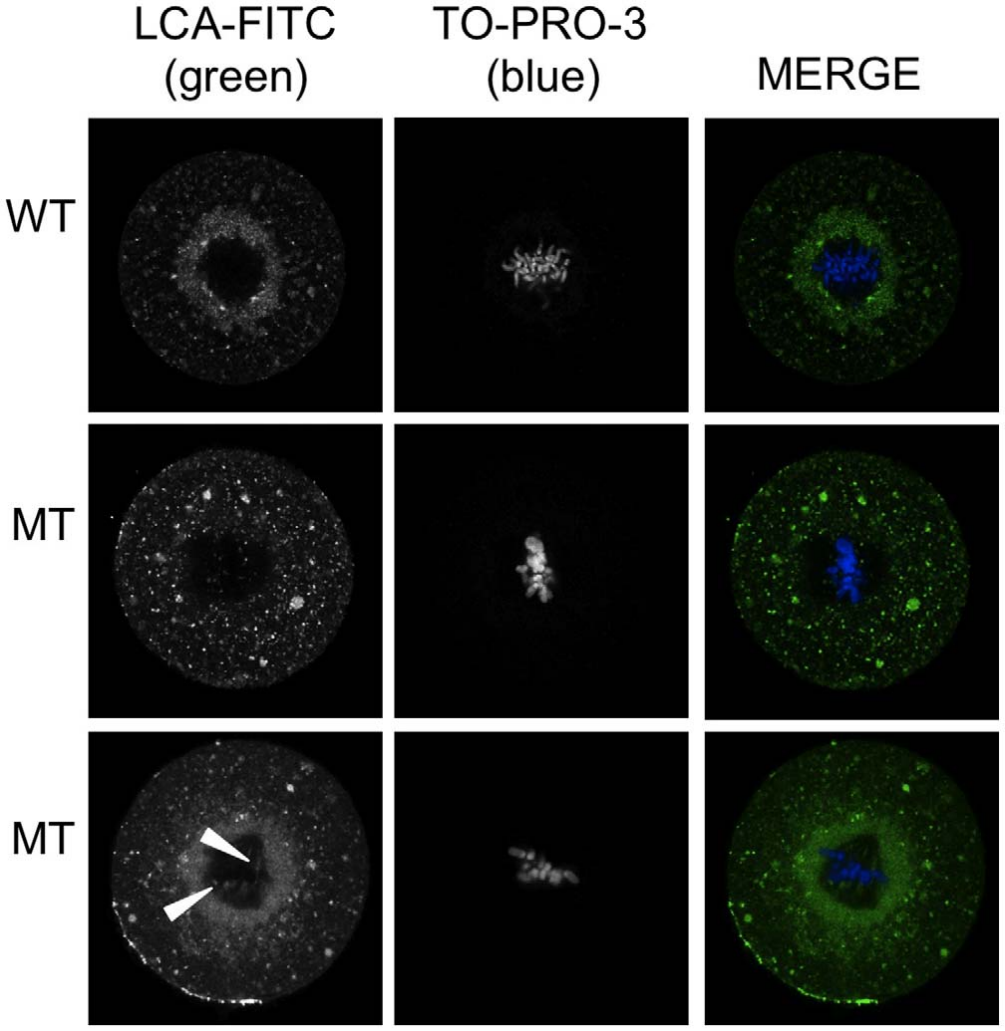
LCA-FITC staining of membranous organelles (ER-nucleus, Golgi apparatus). Representative pictures of WT and MT zygotes at the metaphase stage of the first mitosis stained with LCA-FITC (lens culinaris agglutinin-fluorescein complex). Chromosomes were stained with TO-PRO-3. WT zygotes exhibited a granular LCA positive region surrounding the spindle which was not found in some MT zygotes (shown in upper row). Other MT zygotes displayed some LCA positive granules within the structure of the spindle (shown in lower row, arrowhead).

Very little is known about ER organization during the first mitosis of mouse embryos [13]. To better characterize the morphology of ER compartment, we used a fluorescent marker, LCA-FITC (fluorescent lens culinaris agglutinin-fluorescein complex) which recognizes glycoconjugates located in nuclear envelop, ER and Golgi apparatus [14] (Figure 8). We observed that, in WT zygotes, the compartment stained by LCA-FITC formed a granular layer around the mitotic spindle without invading the structure. In MT embryos, this organization was altered with either absence of the peri-spindle layer and/or persistence of some structures within the spindle (Figure 8, lower panels).

**Figure 9:**
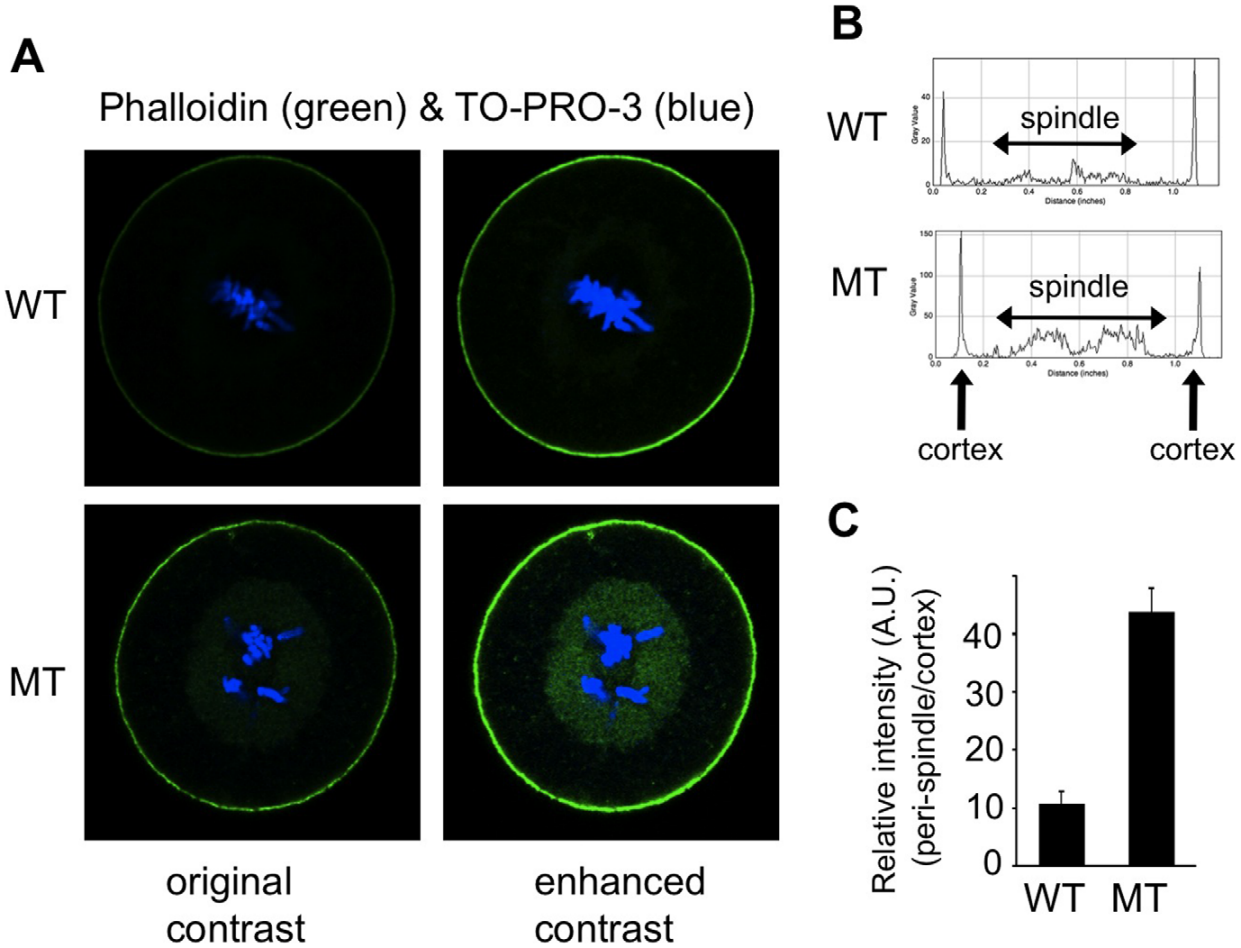
Peri-spindle accumulation of filamentous actin in mutant embryos. (**A**) Representative pictures of WT and MT zygotes at the metaphase stage of the first mitosis stained with phalloidin (green: filamentous actin) and TO-PRO-3 (blue: DNA) shown with original contrast and enhanced contrast to better visualize actin accumulated around the spindle. (**B**) Representative examples of plot profiles (Image J) analyzing the actin staining (normal contrast) along the diameter section of the zygotes. (**C**) Graph showing the relative intensity of actin staining measured at the peri-spindle region and cortex in WT zygotes (n = 5) and MT zygotes (n = 13). Data presented as mean value (A.U.: arbitrary unit). p<0.01.

As it was reported that ER dynamics was depending on actin cytoskeleton in addition to microtubule network during mitosis [15], [16], we stained one-cell embryos with the actin binding phalloidin (Figure 9A). We observed a marked accumulation of filamentous actin surrounding the mutant spindles in comparison to WT ones. We measured the intensity of actin signal and we found that it was 4 times higher in MT zygotes (Figure 9B, C).

All together, our findings indicated that ER organization and probably dynamics were modified in absence of HSP90B1.

## Discussion

Chaperones are important and abundant cellular proteins which protect cells against the consequences of accumulating misfolded proteins. In order to better understand the specific function of some chaperone, gene targeting experiments have been performed (e.g. Hspb1-Hsp25, Hspa1a-Hsp70.1 and Hspa1b-Hsp70.3, Hsp90ab1-Hsp90beta and two ER chaperones, Hsp90b1-Grp94, Hspa5-Grp78) [5]–[7], [17]–[20]. Interestingly and in contrast with cytoplasmic chaperones analyzed so far, deletion of each ER chaperone resulted in early embryonic death. This indicated their importance and non redundant roles during development. However further identification of their function in adult tissues and in particular during gametogenesis remained limited. Conditional knockouts allowed such investigation and our work provides new insights regarding Hsp90b1 in female gametes and early embryos.

Mutant (Zp3-cre; *Hsp90b1^flox/flox^*) females produced oocytes in which the expression of Hsp90b1 was altered from the primary follicular stage. MT oocytes exhibited a thinner zona pellucida. This was probably due to some default in the trafficking of ZP proteins as revealed by the ZP2 positive structures we identified in oocytes from secondary follicles. Those structures were part of the ER network as indicated by a positive staining by HSPA5 antibodies (data not shown). Zona pellucida defects are important even before fertilization as demontrated by ZP1 or Mgat1 knockout which displayed abnormal folliculogenesis, ovulation and severely reduced fertility [21], [22]. In contrast to these knockouts, we did not observe a lower number of ovulation in the conditional deletion of Hsp90b1 but we found a more severe developmental block in comparison to ZP1 mutant embryos that reached the 2-cell stage or Mgat1 ones that were able to implant [21], [22].

One of the main features in the phenotype of mutant Hsp90b1 zygotes, was the high frequency of multipolar spindles. Such anomaly of the spindle was previously reported in cleavage embryos produced by FILIA deficient females but the first mitosis was not affected in the corresponding mutant zygotes [23]. In fact, from the work done by us and others, it appears that HSP90b1 deficiency affects in particular the zygotic mitosis since deficient embryonic cells could be maintained in culture and thus could accomplish cell cycle and mitosis [6]. One possible difference between zygotic mitosis and other mitoses is that during the first cell cycle, there are two nuclear structures, the maternal and paternal pronuclei which form chromosomes that need to be brought together on one single spindle. This situation could be compared to multinucleated cancer cells which generate multipolar spindles [24].

To the best of our knowledge, such role for an ER chaperone during mitosis was never reported and more work is needed to identify the potential clients of HSP90B1 during these very early stages of embryonic development. As potential candidates, an increasing number of genes has been involved in the apparition of multipolar spindle (e.g. Nek7 NIMA related, IKK2, tankyrase1/PARP-a5, EMI1) [25]–[28]. Some of them which are in particular linked to membrane compartments (RINT-1) [29] or spindle matrix (laminB) [30] could be good candidates to interact directly or indirectly with HSP90B1. Nevertheless none of these candidates was reported to interfere with the actin network which seemed to be severely affected in Hsp90b1 mutant zygotes. Recent work demonstrated the important role of actin network in the positioning of the nucleus but there is no available information regarding its participation to spindle formation.

Regarding the ER chaperones themselves, our data were also in agreement with previous work showing that Hspa5 and Hsp90b1 are coregulated [5]. When Hsp90b1 expression was abolished in stem cells, several other ER chaperones: Grp78 (Hspa5), calnexin, calreticulin were upregulated as part of the mechanism of ER stress response [5]. We found that HSPA5 protein was more abundant in mutant oocytes and embryos. Nevertheless the protein profile was not following the transcript profile suggesting two different levels of regulation. Translation of Hspa5 transcripts was reported to be directed by IRES whose activity could be modified by stress [31]. This could explain why HSPA5 protein increased in MT oocytes and embryos. Regarding the higher level of Hspa5 transcripts we observed in MT embryos, we could not find evidence that it was linked to the induction of ER stress response as a consequence of HSP90b1 deficiency. In fact, very little is known regarding ER stress response during those developmental stages, in comparison to numerous studies which investigated the HSF-dependent stress response [32]–[35].

In conclusion, our findings demonstrate that Hsp90b1maternal contribution is involved in ZP protein trafficking during oocyte growth but is not essential to obtain fully grown and MII fertilizable oocytes. In contrast we show that HSP90B1 is critical to complete the first mitosis in murine embryos as its absence provoked a severe disorganization of both the microtubular spindle and the actin–ER network surrounding the spindle. This provides new insights regarding additional HSP90B1 clients whose identification requires further work.

## Materials and Methods

### Ethics Statement

All animal work has been conducted according to relevant national and international guidelines. In particular, protocols for animal breeding and experiments were approved by the Departmental Veterinary Office (Haute-Garonne) according to French legislation (approval ID: 31 09 555 39).

### Transgenic and knockout lines

The *Hsp90b1^flox/flox^* mice were generated previously and are described elsewhere (Hsp90b1^tm1.1Zhli^, MGI:3700024) [7]. They were maintained in a mixed genetic background. To obtain females which produce HSP90B1 deficient oocytes, *Hsp90b1^flox/flox^* mice were crossed with transgenic mice carrying zona pellucida 3 (Zp3) promoter-mediated Cre recombinase (Tg(Zp3-cre)93Knw, MGI:2176187). Zp3-cre transgene is specifically expressed in oocytes in primary and further developped follicles, starting on day 5 after birth [8], [9]. In our experiments, mutant females have the following genotypes: Zp3-cre/+*; Hsp90b1^flox/flox^;* or Zp3-cre/+*; Hsp90b1^flox/del^.* Control females are either wild-type (WT) or *Hsp90b1^flox/flox^*.

### Oocyte and embryo production

Oocytes were collected either at the GV stage from ovarian antral follicles. Embryos were collected at the indicated time from the oviducts following superovulation or not, as indicated. Superovulation, oocytes, embryos collection and culture were performed as previously described [36]. Vaginal plug was used to determine in vivo fertilization and it was considered to be observed on 0.5 dpc (day post coïtum).

### Histology, immunohistochemistry and immunofluorescence

For histology and immunohistochemistry, mouse ovaries were fixed with Bouin, and histological preparations were performed according to a classical procedure (Plateau Technique d'Histopathologie Expérimentale de l'IFR30, Plateforme d'Exploration fonctionnelle/Génopole, Toulouse Midi-Pyrénées). Immunohistochemistry was performed as described before [37]. Antibodies are listed below.

According to the experiment, oocytes or embryos were stained with the following fluorescent dyes: DNA (TOPRO-3, dilution 1∶500 in mounting medium, Invitrogen); actin (phalloidin conjugated either to Alexa 488 or Alexa 546, dilution 1∶40 in M2 medium, Molecular Probes-Invitrogen); ER glycoconjugates (LCA-FITC, solution 100 µg/ml, dilution 1∶10 in M2 medium, Sigma). Fluorescent staining and immunofluorescence protocols were adapted from previous works (TO-PRO-3, phalloidin, LCA-FITC; immunofluorescence [3], [36].

Primary Antibodies used in immunohistochemistry or immunofluorescence were as followed: HSPA5 (rabbit polyclonal, NB100-91794, Novus Biologicals) dilution 1∶50; HSP90b1 (rabbit polyclonal, Ab13509, Abcam) dilution 1∶500 or (rat monoclonal, 9G10, Abcam), dilution 1∶50; FITC-conjugated alpha-tubulin (F 2168, Sigma), dilution 1∶100.

For chromosome spread and DNA staining at the metaphase I stage, oocytes were fixed in 1% PFA [38].

Confocal microscopy and imaging analysis was performed as described previously [3], [36].

### Reverse transcription and quantitative PCR (RTqPCR)

Samples preparation, reverse transcription and quantitative PCR were performed as described in Metchat et al. [3]. Primers used in the present study were as followed: Hspa5: F5′- GAA ATG GCC CAG TGA GAA AA-3′ and R5′- CTT CCA CGT TGC TGA CTT GA-3′; Hsp90b1: F5′- AAC TCG GGA AGA GCC TGA AT-3′ and R5′- GGA GGC GGA ATC TTC TCC A-3′; Ribosomal S16: F5′- AGGAGCGATTTGCTGGTGTGG-3′ and R5′- GCTACCAGGGCCTTTGAGATGGA -3′.

### Statistics

Data were collected from samples collected from at least three animals. The results are presented as means ± SEM as indicated. Student t-test and Chi square were used to perform statistical evaluation, with p<0.05 considered as significant.
